# Digit Ratio (2D:4D; Right-Left 2D:4D) and Multiple Phenotypes for Same-Sex Attraction: The BBC Internet Study Revisited

**DOI:** 10.1007/s10508-023-02703-6

**Published:** 2023-10-17

**Authors:** John T. Manning, Bernhard Fink, Robert Trivers

**Affiliations:** 1 Applied Sports, Technology, Exercise, and Medicine (A-STEM), Swansea University, Swansea, UK; 2 Biosocial Science Information, Biedermannsdorf, Austria; 3 Department of Evolutionary Anthropology, University of Vienna, Djerassiplatz 1, 1030 Vienna, Austria; 4 Human Evolution and Archaeological Sciences (HEAS), University of Vienna, Vienna, Austria; 5 Southfield, St Elizabeth, Jamaica

**Keywords:** Digit ratio, Homosexuality, Prenatal androgen, Sexual attraction, Side differences, 2D:4D

## Abstract

Same-sex attraction may be linked to low prenatal androgen (in men) and high prenatal androgen (in women). Digit ratio (2D:4D) is thought to be a negative correlate of prenatal androgen and right-left 2D:4D (Dr-l) to reflect lateralized differences in sensitivity to prenatal androgen. Lower 2D:4D has been reported for lesbians compared to heterosexuals, but links to high 2D:4D in gay men are less clear. The largest study thus far (the BBC Internet study) found no significant difference between the 2D:4D of lesbians and heterosexual women but a higher 2D:4D in gay men compared to heterosexual men. Here we consider the possibility that low and high prenatal androgen is associated with same-sex attraction in men (*n* = 108,779) and women (*n* = 87,742), resulting in more than two phenotypes. We examined the associations between 2D:4D, Dr-l, and same-sex attraction scores in the BBC Internet study. In contrast to the earlier report, which considered sexual orientation in categories, there were positive linear associations in men (right and left 2D:4D, but not Dr-l) and negative linear associations in women (right 2D:4D and Dr-l, but not left 2D:4D). There were no curvilinear relationships for right and left 2D:4D. However, Dr-l showed a U-shaped association with same-sex attraction in men. Thus, (1) high prenatal androgen may be implicated in female homosexuality, while both low and high prenatal androgen may be implicated in male homosexuality, and (2) large side differences in sensitivity to androgen may be associated with elevated same-sex attraction in men.

## Introduction

Theories about the etiology of homosexuality have focused on the role of prenatal androgens, with the assumption often being that these sex steroids linearly influence sexual orientation. That is, male androphilia is presumed to be associated with low prenatal androgen levels (compared to typical male levels), and female gynephilia is presumed to be associated with high prenatal androgen levels (compared to typical female levels) (for a review, see VanderLaan et al., 2022).

Direct measurement of prenatal sex steroids, particularly levels of 1st-trimester hormones, can be dangerous to the developing fetus. Therefore, many studies that have considered this linear model of sexual orientation have used digit ratio (2D:4D, i.e., the relative lengths of the 2nd and 4th digits; Manning, 2002; Manning et al., 1998). It is thought that 2D:4D is negatively related to prenatal testosterone and positively related to prenatal estrogen (Manning, 2002; Swift-Gallant et al., 2020). Digit ratio is sexually dimorphic (male 2D:4D < female 2D:4D Manning et al., 1998, as is right-left 2D:4D [Dr-l] male Dr-l < female Dr-l Manning et al., 2023). Ossification of the fetal phalanges begins as early as seven weeks (Poznanski, 1974, Chapter 1). Fetal length ratios of the phalanges are established in the 1st trimester of pregnancy and are very similar to ratios seen in children and adults (Garn et al., 1975; Poznanski, 1974, Chapter 2). Early fetal sex differences in 2D:4D have been reported in humans (Galis et al., 2010; Malas et al., 2006) and mice (Zheng & Cohn, 2011). The sex difference in 2D:4D is largely independent of digit growth in children and teenagers (Manning et al., 1998; Trivers et al., 2006, 2020). It is found across ethnic groups (Butovskaya et al., 2021; Manning, 2002) and is not influenced by digit length allometry (Butovskaya et al., 2021; Jagetoft et al., 2022; Manning & Fink, 2018; Skorska et al., 2021). Manipulation of fetal sex steroids in rodents has shown that sexual dimorphism of 2D:4D is determined by a balance of prenatal testosterone-to-estrogen in an early and narrow developmental window (Auger et al., 2013; Talarovicová et al., 2009; Zheng & Cohn, 2011).

Thus, there is considerable evidence that 2D:4D is a correlate of prenatal testosterone and estrogen. However, one aspect of the sex difference in 2D:4D, i.e. its effect size, has been the focus of debate. Digit ratios calculated from self-reported digit lengths have sex differences (male < female) with low effect sizes of about *d* = 0.20 for 2D:4D and *d* = 0.07 for Dr-l (Manning et al., 2007). Experimenter-measured digit lengths from photocopies or scans yield 2D:4Ds with medium sex differences of approximately *d* = 0.50 and *d* = 0.30 for Dr-l (Manning, 2002; Manning et al., 2000). In contrast, it is reported that in male compared to female fetuses, concentrations of testosterone in the prenatal gonads are about 15 times as large (Reyes et al., 1973) and in the serum 4 to 5 times as large (Reyes et al., 1974). Testosterone levels in amniotic fluid also show higher concentrations in males compared to females with effect sizes of *d* ≥ 1.5 (Knickmeyer et al., 2005; Lutchmaya et al., 2004). The apparent discrepancy between the magnitude of sex differences in 2D:4D and that of fetal testosterone levels has been discussed by Manning and Fink (2023). In male fetuses, testosterone levels peak in the 2nd trimester. It is then that sex differences in testosterone concentration are at their highest. Many measurements of testosterone levels have been reported from the 2nd trimester. In contrast, there is evidence that variation in 2D:4D is more or less fixed in the 1st trimester. Measurement landmarks of 2D:4D such as flexion creases and the bone-to-bone ratios of the phalanges are established by the end of the 1st trimester, and the marked 2nd-trimester changes in testosterone are not reflected in 2nd-trimester sexual dimorphism of 2D:4D or mean 2D:4D (Manning & Fink, 2023). In comparison to 2nd-trimester sexual dimorphism, sex differences in testosterone levels are likely to be weak in the 1st trimester as fetal testes are small (Reyes et al., 1973) and other non-dimorphic sources of androgen such as maternal sex steroids may assume greater importance (Baxter et al., 2019; for a review, see Manning & Fink, 2023).

In common with 2D:4D, Dr-l is also sexually dimorphic and the magnitude of the dimorphism is similar across ethnic groups (Manning et al., 2022b, 2023). Effect sizes for correlations between 2D:4D and target traits such as spermatogenesis are often larger for right 2D:4D compared to left 2D:4D (e.g., Manning et al., 1998). Moreover, right 2D:4D is more sexually dimorphic than left 2D:4D (Hönekopp & Watson, 2010). Right-left differences in 2D:4D correlate with many sex-dependent traits suggesting Dr-l is negatively related to prenatal testosterone (Manning, 2002). Of particular relevance to sexual orientation is the association between Dr-l and hand preference such that low Dr-l is associated with left-handedness (in a peg test, Manning et al., 2000; writing hand preference, Manning & Peters, 2009).

Comparisons of 2D:4D between lesbians and female heterosexuals have reported the former have lower 2D:4D (i.e., they are androgenized) than the latter (for meta-analysis see Grimbos et al., 2010). Failure to replicate low 2D:4D in female gynephilia has more recently been reported. However, such studies have considered effects in samples of *n* < 100 (Holmes et al., 2021; Kangassalo et al., 2011; Xu et al., 2019). The links between 2D:4D and male sexual orientation appear to be more complex. In comparison to heterosexuals, studies have shown gay men to have lower 2D:4D (Robinson & Manning, 2000), equal 2D:4D (Williams et al., 2000), and higher 2D:4D (Manning et al., 2007). Unsurprisingly, a meta-analysis has shown no overall difference in 2D:4D between heterosexual and gay men (Grimbos et al., 2010; more recent findings of high 2D:4D in gay men in comparison to heterosexuals have reported relatively small samples of *n* < 100 participants Kangassalo et al., 2011; Xu et al., 2019). One explanation for these meta-analytical findings is that there is a curvilinear relationship between prenatal androgen and sexual orientation in men, i.e., male androphilia is associated with very low and very high prenatal androgen. In contrast, prenatal androgen may be linearly related to female sexual orientation, i.e., female gynephilia may be linked to high levels of prenatal androgen only. Reports of sexual orientation data from the BBC Internet study (for a description, see Reimers, 2007) have shown that in comparisons with heterosexuals, 2D:4D is higher in gay men and does not differ in lesbians (Manning et al., 2007). These analyses used categorical (heterosexual/homosexual/bisexual) data for sexual orientation. However, the BBC Internet study also contains another item concerning sexual attraction scores towards men and women. Here we consider linear and curvilinear relationships between 2D:4D (right-hand 2D:4D; left-hand 2D:4D and the difference of right-left 2D:4D [Dr-l]) and same-sex attraction scores. The meta-analytic finding by Grimbos et al. (2010) of no significant association between 2D:4D and sexual orientation in men was based largely on the assumption of a negative linear relationship between prenatal testosterone and male androphilia. The suggestion that both higher or lower prenatal testosterone is linked to male same-sex preference appears to have originated in experimental studies of rodents (e.g., Dela Cruz & Pereira, 2012; Diamond et al., 1973; Henley et al., 2010; Piacsek & Hostetter, 1984; Zadina et al., 1979). In humans, a number of researchers have discussed the possibility of a curvilinear relationship between prenatal androgens and same-sex attraction in men (e.g., Skorska & Bogaert, 2017; Swift-Gallant, 2019).

Studies on handedness indicate a more complex curvilinear picture with extremes of left- and right-handedness reported among gay men (Bogaert, 2007; Ellis et al., 2017; Kishida & Rahman, 2015; Lalumière et al., 2000). Thus, both feminization and masculinization (i.e., low and high prenatal testosterone) may be linked to male androphilia. The finding of low and high 2D:4D linked to “top” and “bottom” roles respectively for anal sex may also map onto a curvilinear relationship between prenatal testosterone and male androphilia (Swift-Gallant et al., 2021). Concerning female gynephilia, there appears to be no evidence for or against extremes of handedness. That is, this question has been largely neglected. However, there is some support for links between low and high 2D:4D and butch and femme roles respectively (Brown et al., 2002).

The purpose of this study was to examine the types of relationships (linear vs. curvilinear) between 2D:4D variables (right and left 2D:4D, and Dr-l) and same-sex attraction scores in the BBC Internet study. Many reports of digit ratio and same-sex attraction have considered right and left 2D:4D but have not considered Dr-l. There are associations between same-sex attraction and handedness and Dr-l and handedness in the literature. Therefore, we place some emphasis on this variable in addition to the more conventional right and left hand 2D:4D. We hypothesized that (1) a positive association between the digit ratio variables and male same-sex attraction, and a negative relationship between the digit ratio variables and female same-sex attraction would suggest a linear influence of prenatal testosterone on same-sex attraction, whereas (2) a U-shaped relationship between the digit ratio variables and same-sex attraction scores would suggest a curvilinear relationship of prenatal testosterone on same-sex-attraction, with both low and high prenatal androgen implicated in male and female non-heterosexuality.

## Method

The current study used data from a large online survey, i.e., the BBC Internet study—a multiethnic and multinational survey, hosted by the BBC Science and Nature website in July 2005. Details of the BBC Internet study, including information about the general methodology, ethical considerations, anonymity, and confidentiality are given by Reimers (2007); see also the archived URL: http://www.bbc.co.uk/science/humanbody/sex.

### Participants

There were 255,116 (52.7% males) participants who completed the study. These included individuals of ethnic groups: 84.1% White, 6.3% Asian or Asian British, 2.2% Chinese, 1.2% Middle or Near Eastern, 0.8% Black or Black British, 0.8% as Black other, and the remainder (2.2%) of mixed origin. The largest proportion of respondents lived in the UK (46.8%) and the USA (27.7%).

### Procedure

The study comprised six sections containing a total of about 200 questions, including an item that asked for the sexual orientation of the participant. Participants were asked to measure their index finger and ring finger on the palm of the right and left hand and report their measurements on a drop-down menu from 10 to 100 mm. The item was worded as follows: “For this task, you will need a ruler. Hold your right hand in front of you. Look at where your ring finger joins the palm of your hand. Find the bottom crease. Go to the middle of this crease. Put the 0 of your ruler exactly in the middle of the bottom crease. Make sure the ruler runs straight up the middle of your finger. Measure to the tip of your finger (not your nail) in millimeters. Every millimeter counts, so it is important to do this as accurately as possible. You are contributing to real scientific research and it will determine your results.”

### Measures

An item on sexual attraction was worded as follows: “How sexually attracted are you to …?” with options “men” and “women”, both on a 7-point scale (1 = not at all, 7 = very).

Digit lengths were self-reported. As in previous reports of the BBC study (Manning et al., 2007, 2022a, 2022b, 2023), we restricted our analyses to participants with 2D:4D in the right and left hands within the range of 0.80–1.20. We did this because finger lengths that are self-measured occasionally include measurement errors that result in extreme values of 2D:4D outside the range of 0.80–1.20 (e.g., Manning et al., 2007). Considering sexual dimorphism in the total BBC sample showed that self-reported finger lengths gave significant dimorphism for the right and left hand but with very low effect sizes of *d* = 0.09 for the right hand and *d* = 0.06 for the left hand. After excluding outliers of 2D:4D, the effect sizes increased somewhat to *d* = 0.20 and *d* = 0.15 for right and left 2D:4D, respectively.

A comparison of 2D:4D from self-reported digit lengths and experimenter-measured digit lengths from photocopies has reported high means and high *SD*s in the former compared to the latter (Caswell & Manning, 2009, *n* = 329 [77 males]). There were a small number of extreme values (1%) which presumably arose from random errors. Restricting 2D:4D to the range of 0.8–1.20 led to increasing correlations between self-reported and experimenter 2D:4D (right 2D:4D *r* = 0.52, left 2D:4D *r* = 0.41, both *p* < 0.0001). It also reduced the *SD*s and increased the sex difference in self-reported 2D:4D (right hand: males 0.982 [0.037], females 0.989 [0.045], *d* = 0.17; left hand: males 0.977 [0.047], females 0.979 [0.046], *d* = 0.04). The 2D:4D from photocopies showed the expected sex difference but here the effect size was higher (right hand: males 0.967 [0.032], females 0.979 [0.033], *d* = 0.40; left hand: males 0.950 [0.030], females 0.963 [0.035], *d* = 0.40). Note, because the self-reported 2D:4Ds are calculated from direct digit measurements, their means are lower than those from indirect photocopy measurements. Caswell and Manning (2009) concluded that self-measurements directly on the fingers are likely to include a small percentage of major errors which result in outlier values of 2D:4D. When these are excluded, there are weak but significant associations between 2D:4D calculated from self-measurements and 2D:4D from rater-reported measurements. In addition, many minor “errors” in self-measurements probably result in a reduction in the effect size of the sexual dimorphism of 2D:4D, and in effect sizes for correlations for 2D:4D with target variables. These limitations can be countered if large numbers of participants are recruited.

## Results

### Descriptive Statistics

**Fig. 1:**
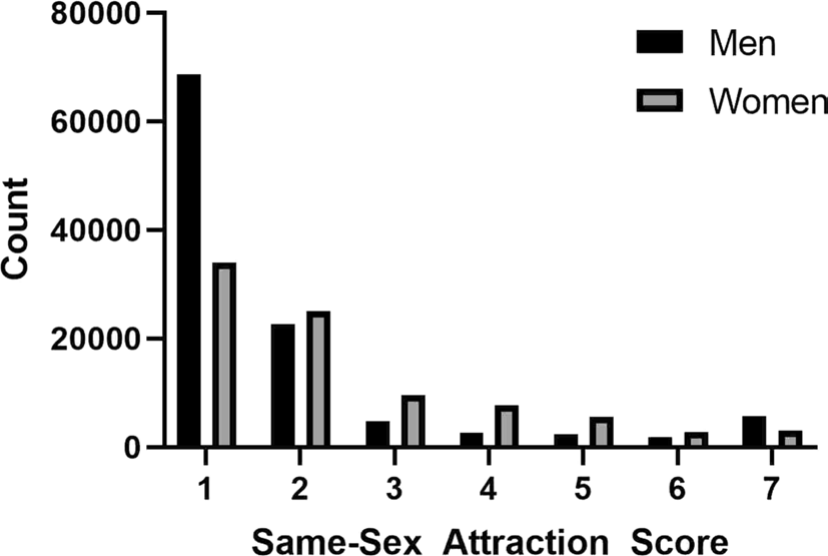
Same-sex attraction scores for men (n = 108,779) and women (n = 87,742)

Same-sex attraction scores were reported by 108,779 men and 87,742 women. The mean (*SD*) male score was 1.86 (1.59) with a range from 1 (*n* = 68,600) to 7 (*n* = 5720) and the mean female score was 2.39 (1.62) with a range from 1 (*n* = 33,949) to 7 (*n* = 3098) (Fig. 1).

Table 1 reports the untransformed values of skewness and kurtosis for same-sex attraction scores, right and left 2D:4D and right-left 2D:4D (Dr-l). For both sexes, the same-sex attraction scores showed high values of skewness and kurtosis. A log(x) transformation of the same-sex scores reduced skewness for both men [mean 0.17 (0.25), skewness 1.35, kurtosis 0.70] and women [mean 0.29 (0.27), skewness 0.40, kurtosis − 1.05]. In all subsequent analyses, we report results for both non-transformed and log(x) transformed same-sex attraction scores.

**Table 1:** Untransformed values of skewness and kurtosis for sexual attraction scores, right and left 2D:4D, and right-left 2D:4D (Dr-l) in men and women

	Men		Women	
	Skewness	Kurtosis	Skewness	Kurtosis
Same-sex attraction	2.206	3.932	1.251	0.743
Right 2D:4D	0.535	1.706	0.539	1.599
Left 2D:4D	0.496	1.832	0.558	1.853
Dr-l	0.115	8.899	0.157	6.769

The means (*SD*s) for digit ratio variables were as follows: Right 2D:4D, men 0.984 (0.049), women 0.994 (0.051); Left 2D:4D, men 0.984 (0.048), women 0.992 (0.049), and Dr-l, men − 0.001 (0.040), women 0.002 (0.043). Digit ratio variables showed low values of skewness (Table 1) and were, therefore, not log(x) transformed.

### Regression Analyses

A series of linear and curvilinear (2nd-order polynomial) regression analyses split by sex, respectively, were performed (Table 2). Dependent variables were same-sex attraction scores [non-transformed and log(x) transformed] split by sex. Independent variables were right 2D:4D, left 2D:4D, and Dr-l.

**Table 2:** Relationships between same-sex attraction scores and digit ratio variables in men and women

Trait	Linear regression					2nd order polynomial				
	B	SE B	β	*t*	*p*	B	SE B	β	*t*	*p*
*Men*										
R2D:4D	.437 (.050)	.098 (.016)	.013 (.010)	4.44 (3.145)	** < .0001** **(.002)**	− 1.21 (− .442)	1.080 (.175)	− .074 (− .168)	1.117 (2.521)	.26 **(.01)**
L2D:4D	.530 (.071)	.101 (.016)	.016 (.013)	5.229 (4.294)	** < .0001** **(< .0001)**	− 1.84 (− .532)	1.124 (.182)	− .109 (− .195)	1.633 (2.915)	.10 **(.004)**
Dr-l	− .091 (− .024)	.120 (.019)	− .002 (− .004)	.759 (1.240)	.448 (.22)	2.583 (.341)	.902 (.140)	.009 (.007)	2.863 (2.329)	**.004** **(.02)**
*Women*										
R2D:4D	− .313 (− .066)	.107 (.018)	− .010 (− .012)	2.914 (3.676)	.**004** **(.0002)**	1.403 (− .159)	1.152 (.192)	.089 (− .061)	1.218 (.828)	.22 (.41)
L2D:4D	− .099 (− .024)	.112 (.019)	− .003 (− .004)	− .887 (1.260)	.38 (.21)	.536 (− .321)	1.211 (.202)	.033 (− .177)	.442 (1.588)	.66 (.11)
Dr-l	− .311 (− .062)	.127 (.021)	− .008 (− .010)	2.446 (2.925)	**.02** **(.003)**	2.514 (.148)	.996 (.166)	.009 (.003)	2.525 (.893)	**.01** (.37)

Relationships with log(x) transformed same-sex attraction scores are given in parenthesis. The *p* values are highlighted where associations were significant between digit ratio variables and both untransformed and transformed same-sex attraction scores

#### Linear Regression Analyses

**Fig. 2:**
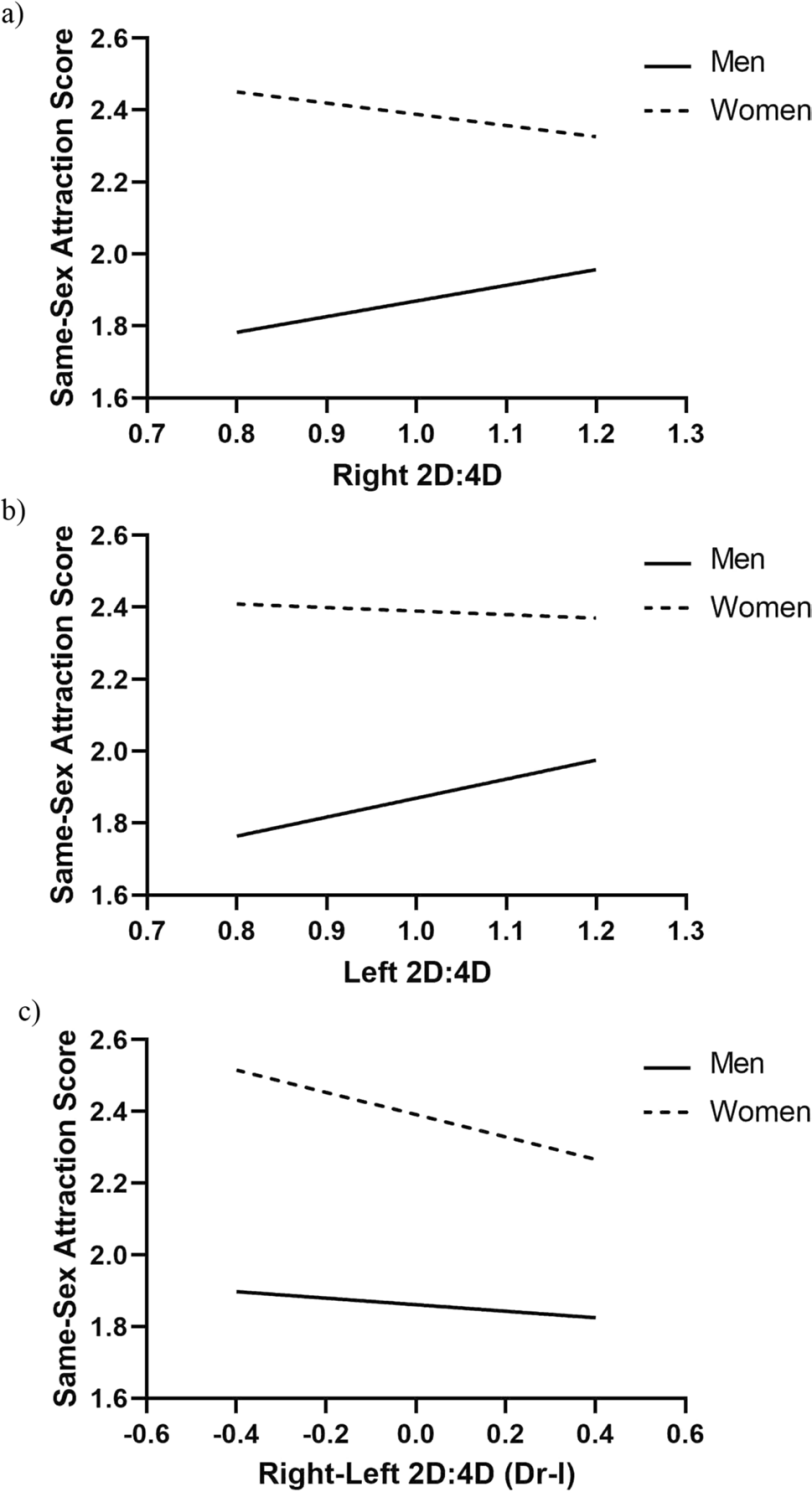
Linear regressions for men’s and women’s untransformed same-sex scores on **a** right 2D:4D [men *p* < .0001, women *p* = .004], **b** left 2D:4D [men *p* < .0001, women *p* = .38], and **c** right-left 2D:4D (Dr-l) [men *p* = .448, women* p* = .02]

For linear relationships, there were positive associations between 2D:4D (right and left) and same-sex scores for males and negative relationships between right 2D:4D and Dr-l and same-sex scores for females. This result was obtained with both untransformed and log(x) transformed same-sex scores. Details of the associations are as follows:

Male right and left 2D:4D were positively related to both non-transformed same-sex attraction scores (right 2D:4D: y = 1.432 + 0.437*x, β = 0.013, *p* < 0.0001; left 2D:4D: y = 1.339 + 0.53*x, β = 0.016, *p* < 0.0001) and to log(x) transformed same-sex attraction scores (right 2D:4D: y = 0.123 + 0.05*x, β = 0.010, *p* < 0.0001; left 2D:4D: y = 0.102 + 0.071*x, β = 0.013, *p* < 0.0001). There were no significant relationships for Dr-l (Fig. 2a–c).

Women showed a negative relationship between right 2D:4D and both non-transformed (y = 2.701 − 0.313*x, β = − 0.010, *p* < 0.01) and log(x) transformed same-sex attraction scores (y = 0.355 − 0.066*x, β = − 0.012, *p* < 0.001). There was no significant relationship for left 2D:4D. In addition, there was a negative association between Dr-l and non-transformed same-sex attraction scores (y = 2.391 − 0.311*x, β = − 0.008, *p* < 0.05) and log(x) transformed same-sex attraction scores (y = 0.29 − 0.062*x, β = − 0.010, *p* < 0.01) (Fig. 2a–c).

#### Curvilinear Regression Analyses

There were no significant 2nd-order polynomial associations between 2D:4D (right and left) and non-transformed same-sex attraction scores in men. For log(x) transformed same-sex attraction scores, there were significant (inverted U-shaped) relationships with both right 2D:4D (y = − 0.316 + 0.931*x − 0.442*x^2^, β = − 0.168, *p* < 0.01) and left 2D:4D (y = − 0.424 + 1.13*x − 0.532*x^2^, β = − 0.195, *p* < 0.01). These curvilinear associations were not stronger than those for linear associations with 2D:4D (right and left) and same-sex attraction scores. Dr-l and same-sex attraction scores showed curvilinear U-shaped relationships that were significant for both non-transformed (y = 1.857 − 0.1*x + 2.583*x^2^, β = 0.009, *p* < 0.01) and log(x) transformed (y = 0.171 − 0.025*x + 0.341*x^2^, β = 0.007, *p* = 0.05).

**Fig. 3:**
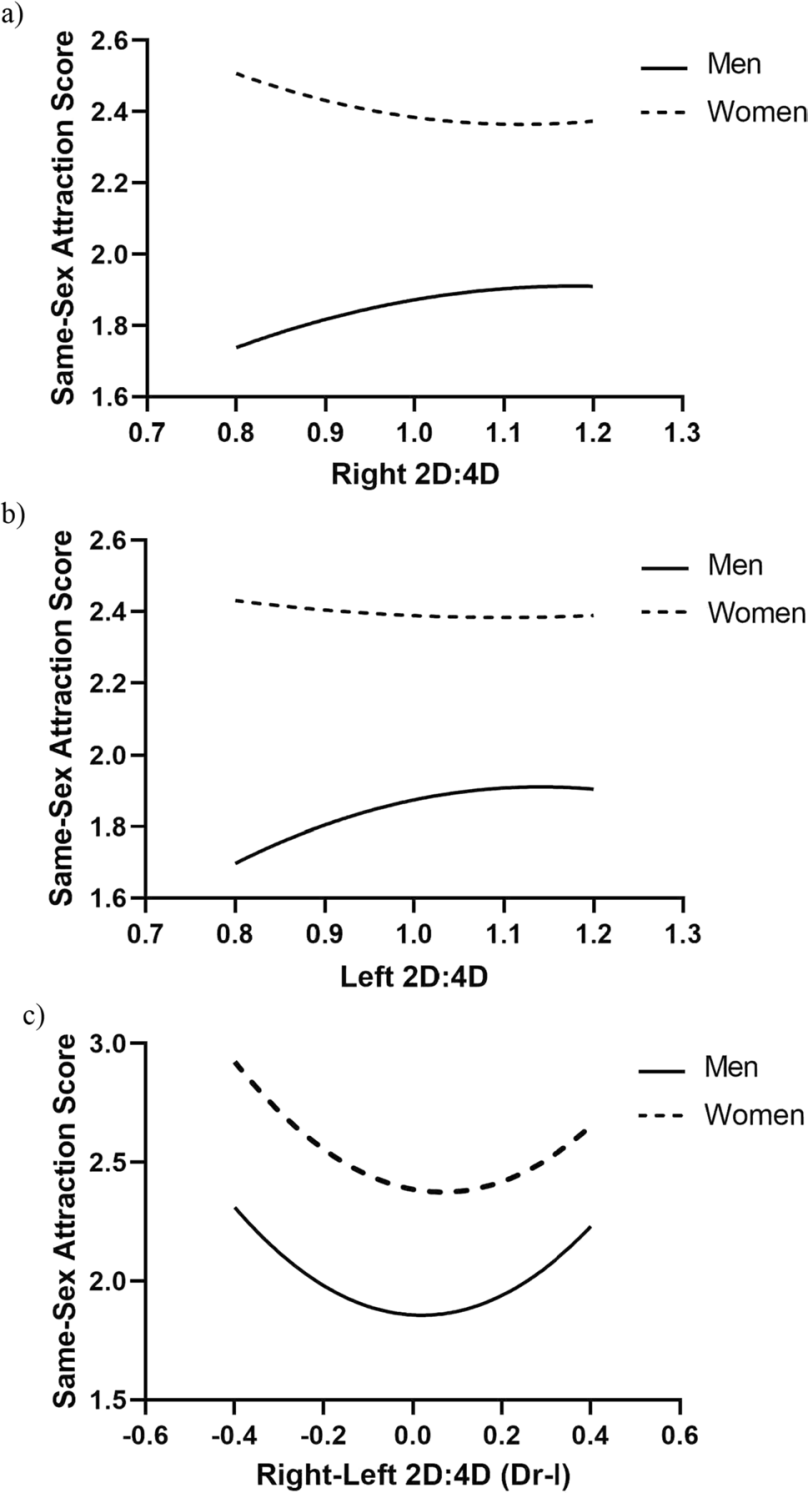
Curvilinear regressions (2nd-order polynomial) for men’s and women’s untransformed same-sex scores on **a** right 2D:4D [men *p* = .26, women *p* = .22], **b** left 2D:4D [men *p* = .10, women *p* = .66], and **c** right-left 2D:4D (Dr-l) [men *p* = .004, women *p* = .01]

There were no significant associations between digit ratios (right or left) and same-sex attraction scores (untransformed or transformed) in women. The relationship between Dr-l and untransformed same-sex attraction scores was U-shaped and stronger than the linear relationship for these variables. The association between Dr-l and log(x) transformed same-sex attraction scores was not significant (Fig. 3a–c).

## Discussion

We have found positive linear relationships between 2D:4D (right and left hands) and same-sex attraction scores in male respondents of the BBC Internet study. The associations were statistically significant for both non-transformed and log(x) transformed same-sex attraction scores. Our finding of significant relationships for both non-transformed and log(x) transformed same-sex attraction scores suggests these are robust associations. There were also curvilinear associations between 2D:4D (right and left hands) and log(x) transformed scores only, but these relationships were weaker than their respective linear associations and were of the inverted U-shaped form. There was no linear relationship between Dr-l and non-transformed or log(x) transformed same-sex attraction scores. However, there were curvilinear associations (U-shaped) between Dr-l and both non-transformed and log(x) transformed same-sex attraction scores.

Both right 2D:4D and Dr-l showed negative linear relationships with same-sex attraction scores in women. The associations were significant for both non-transformed and log(x) transformed same-sex attraction scores. There was no curvilinear relationship between right 2D:4D and same-sex attraction. A pattern of significant associations between right 2D:4D but not left 2D:4D and target traits is common in digit ratio studies. This suggests the correlation is real. In addition to the negative linear relationship with same-sex scores, there was also a U-shaped association between Dr-l and non-transformed (but not log[x] transformed) same-sex scores.

Overall, the linear associations between right and left 2D:4D and sexual male/female attraction scores in the BBC Internet study support a two-phenotype model of same-sex attraction. That is, male androphilia is associated with low prenatal androgen levels (compared to male norms) and female gynephilia is associated with high prenatal androgen levels (compared to female norms). More specifically, our finding of positive associations for 2D:4D (right and left hands) and male same-sex attraction scores is consistent with an etiology of low prenatal testosterone/high prenatal estrogen influences in male androphilia. In this regard, our results map onto the categorical (heterosexual; homosexual; bisexual) data from the BBC Internet study reported by Manning et al. (2007). We have found a negative correlation between right 2D:4D and female same-sex attraction. This is in contrast to the earlier report of no significant difference in 2D:4D between lesbians and heterosexual women (Manning et al., 2007). We feel our current finding goes some way to resolving the apparent discrepancy between the BBC Internet study findings and those studies that reported masculinized 2D:4D in lesbians (e.g., Brown et al., 2002). Thus, our 2D:4D results for females support an etiology of high prenatal testosterone/low prenatal estrogen influence in female gynephilia.

In contrast to our 2D:4D findings suggesting a linear two-phenotype model for same-sex attraction, a consideration of Dr-l yielded curvilinear relationships indicating three phenotypes for same-sex attraction. That is, male androphilia is associated with very low and very high prenatal androgen, suggesting two phenotypes. In contrast, prenatal androgen may be linearly related to female sexual orientation, suggesting one phenotype. More specifically, we observed a U-shaped association between Dr-l and male same-sex attraction scores (both non-transformed and log[x] transformed). There is evidence that Dr-l is negatively related to androgen levels generated under challenge (Crewther et al., 2015; Kilduff et al., 2013), to free background levels of androgens (Banyeh et al., 2023) and it is negatively associated with early prenatal maternal androgen in a New World primate (Titi monkeys; Baxter et al., 2019). In humans, it is sexually dimorphic (male Dr-l < females Dr-l) and the dimorphism appears to be stable, at least in adolescents and adults (Manning et al., 2023). If 2D:4D and Dr-l are both negatively related to prenatal testosterone, why is same-sex attraction related linearly to the former but curvilinearly to the latter? We may have here an example of sex-dependent effects which are dependent on the side (left/right).

Male-typical traits, such as low 2D:4D and testis size tend to be expressed more intensely on the right side and female-typical traits, such as high 2D:4D and breast size, are more often found on the left side (Kimura, 1994; Manning, 2002, pp. 21–23; Tanner, 1990). These morphological asymmetries have been linked to sex-dependent cognitive abilities (Kimura, 1994). The current study finds that men with low right 2D:4D relative to left 2D:4D or high right 2D:4D relative to left 2D:4D report high same-sex attraction scores. Hence, this can be interpreted as evidence of a link between male androphilia and low or high prenatal testosterone. This may map to the finding of high rates of left-hand and extreme right-hand preference in male androphilia (Bogaert, 2007; Ellis et al., 2017; Kishida & Rahman, 2015; Lalumière et al., 2000).

Concerning female gynephilia, there was a similar U-shaped curve for the regression of non-transformed same-sex attraction scores on Dr-l. We suggest caution in interpreting this relationship as we did not find it with log(x) transformed scores. However, there is some support for a U-shaped relationship between same-sex attraction scores and prenatal testosterone. Manning et al. (2022b) have reported a negative relationship between parental income and the 2D:4D of their adult male and female children. That is, low-parental-income families tend to have feminized children whereas children of high-income families are androgenized. This finding may be a Trivers-Willard effect such that low-income (low-condition) mothers estrogenize their children in order to maximize the fitness of their daughters. In contrast, high-income (high-condition) mothers androgenize their children in order to maximize the fitness of their sons. The negative association between 2D:4D and family income led to a study concerning parental income and same-sex attraction. Curvilinear relationships were found such that, in comparison to children from average-income parents, the children from low-income and high-income households reported elevated rates of same-sex attraction in both sexes (Manning et al., 2022a). These U-shaped relationships provide further support for a relationship between low and high testosterone or high estrogen and male androphilia and female gynephilia.

Can a model of the relationship between same-sex attraction and high prenatal sex steroids (both testosterone and estrogen) help to explain the common occurrence of male androphilia and female gynephilia? It is unlikely that non-heterosexuality is maintained by recurrent mutation. The substantial direct fitness losses associated with same-sex attraction are incompatible with such a model (Apostolou, 2022). Indirect fitness effects of kin selection may offset some of the direct losses in fitness (VanderLaan & Vasey, 2016; VanderLaan et al., 2016; Vasey & VanderLaan, 2014). However, we suggest another model for the evolutionary origins of same-sex attraction which acts through selection on maternal manipulation of fetal sex steroids rather than the same-sex attraction itself.

We consider a model that represents a modified version of the Trivers-Willard hypothesis (Manning et al., 2022a, 2022b; Trivers & Willard, 1973). The model assumes that (1) the variance in direct fitness for males is greater than that for females (see Bateman, 1948), (2) variation in maternal condition (e.g., in response to resource availability; Mathews et al., 2008 or maternal stress; Ward & Weisz, 1980) affects fetal sex steroids, (3) this leads to fitness consequences in the offspring. Low-condition mothers (e.g., those from low-income families) will then be selected to feminize their fetuses to maximize the fitness of female offspring. High-condition mothers (e.g., those from high-income families) will be selected to masculinize their fetuses to maximize the fitness of their male offspring. High prenatal estrogen in low-condition mothers and high prenatal testosterone in high-condition mothers are then associated with elevated same-sex attraction scores when the maternal condition is very low or very high. Thus, non-heterosexuality in offspring arises from selection pressures on mothers to maximize the fitness of their daughters when the maternal condition is low and their sons when the maternal condition is high. Such a model represents a modified version of the Trivers-Willard hypothesis.

In conclusion, the present study found linear relationships between 2D:4D and same-sex attraction scores, which support a two-phenotype model for male androphilia and female gynephilia. That is, high same-sex attraction scores are associated with high 2D:4D (low prenatal testosterone/high prenatal estrogen) for the former and low 2D:4D (high prenatal testosterone/low prenatal estrogen) for the latter. However, when considering right-left side effects on 2D:4D (Dr-l) there were curvilinear effects: same-sex attraction scores were greatest for both low Dr-l and high Dr-l. These U-shaped effects were strongest for male androphilia. They suggest, for men, a two-phenotype model for non-heterosexuality such that same-sex attraction is associated with both high prenatal estrogen and high prenatal testosterone. For women, the data reported here support a one-phenotype model for non-heterosexuality with an association between same-sex attraction and high prenatal testosterone.

## Data Availability

The data are available upon reasonable request from the first author.
